# Morphological, Photosynthetic, and Physiological Responses of Rapeseed Leaf to Different Combinations of Red and Blue Lights at the Rosette Stage

**DOI:** 10.3389/fpls.2016.01144

**Published:** 2016-08-03

**Authors:** Chang Shengxin, Li Chunxia, Yao Xuyang, Chen Song, Jiao Xuelei, Liu Xiaoying, Xu Zhigang, Guan Rongzhan

**Affiliations:** College of Agriculture, Nanjing Agricultural UniversityNanjing, China

**Keywords:** red and blue LEDs, *Brassica napus* L., net photosynthetic rate, PSII photodamage, stress responses

## Abstract

Rapeseed (*Brassica napus* L.) is sensitive to light quality. The factory production of rapeseed seedlings for vegetable use and for transplanting in the field requires an investigation of the responses of rapeseed to light quality. This study evaluated the responses of the leaf of rapeseed (cv. “Zhongshuang 11”) to different ratios of red-photonflux (RPF) and blue-photonflux (BPF) from light emitting diodes (LEDs). The treatments were set as monochromatic lights, including 100R:0B% and 0R:100B%, and compound lights (CLs), including 75R:25B%, 50R:50B%, and 25R:75B%. The total photonflux in all of the treatments was set as 550 μmolm^−2^s^−1^. With an increase of BPF, the rapeseed leaves changed from wrinkled blades and down-rolled margins to flat blades and slightly up-rolled margins, and the compact degree of palisade tissue increased. One layer of the cells of palisade tissue was present under 100R:0B%, whereas two layers were present under the other treatments. Compared to 100R:0B%, 0R:100B% enhanced the indexes of leaf thickness, leaf mass per area (LMA), stomatal density, chlorophyll (Chl) content per weight and photosynthetic capacity (*P*_max_), and the CLs with high BPF ratios enhanced these indexes. However, the 100R:0B% and CLs with high RPF ratios enhanced the net photosynthetic rate (*P*_n_). The leaves under the CLs showed growth vigor, whereas the leaves under 100R:0B% or 0R:100B% were stressed with a low *F*_v_/*F*_m_ (photosynthetic maximum quantum yield) and a high content of ${\text{O}}_{2}^{.-}$ and H_2_O_2_. The top second leaves under 100R:0B% or 0R:100B% showed stress resistance responses with a high activity of antioxidase, but the top third leaves showed irreversible damage and inactivity of antioxidase. Our results showed that the rapeseed leaves grown under 0R:100B% or CLs with a high BPF ratio showed higher ability to utilize high photonflux, while the leaves grown under 100R:0B% or CLs with a low BPF ratio showed higher efficiency in utilizing low photonflux. Under different R:B photonflux ratios, red and blue lights may play mutual roles in *P*_n_. When the blue light dominated, the *P*_n_ showed a B-preference. When the red light dominated, the *P*_n_ showed an R-preference. Furthermore, CLs were suitable for the *P*_n_ of rapeseed seedlings.

## Introduction

Red (600–700 nm) and blue (400–500 nm) spectra are the two most attractive spectrum wavelengths for plants due to their important function in plant growth and development. Red light can excite the biologically inactive phytochrome Pr form into a biologically active Pfr form, which has maximum absorbance in far-red (FR) light (Li et al., 2011). Blue light can stimulate the activities of cryptochrome and phototropin (Inoue et al., 2010; Yu et al., 2010). The excited state of photosynthetic pigments is accumulated by blue light absorption and rapidly relaxes through heat loss to an energy level which is accessed by red light absorption and is the effective threshold for energy storage (Blankenship et al., 2011). The effects of 100R:0B% or 0R:100B% for plant growth are species specific. 100R:0B% enhances the stem/hypocotyl elongation of lettuce (Hirai et al., 2006) and *Arabidopsis* (Zhao et al., 2007). For eggplant (Hirai et al., 2006), petunia (Fukuda et al., 2016) and cucumber (Hernández and Kubota, 2016), this effect is the opposite. In the compound lights (CLs), a low ratio of blue-photonflux (BPF) increased the stem/hypocotyl elongation and leaf expansion in soybeans, radishes, wheat and cucumbers, whereas the high ratio of BPF resulted in more compact plants (Cope and Bugbee, 2013; Hernández and Kubota, 2016). However, there are few studies on the response of the leaf shape and the anatomical structure response to the combination of red and blue LEDs.

Hogewoning et al. (2010) found that the leaves of cucumbers under 100R:0B% displayed a dysfunctional photosynthetic operation, whereas the leaves under 0R:100B% had a low photosynthetic ability but also showed healthy and functional photosynthesis. Along with the spectra ratio change from 100R:0B% to 50R:50B%, the *P*_max_, LMA, Chl content, photosynthetic N use efficiency, and the Chl:N ratio of cucumber leaves showed an increase with the dose. Hernández and Kubota (2016) found that the Chl content, *P*_n_, and stomatal conductance increased with the spectra ratio change from 100R:0B% to 0R:100B%. In previous experiments, we found some different trends from those results above, especially in the serious stress of the leaf under 0R:100B%.

Rapeseed is the second largest oil crop in the world, and China ranks second in the world in the production of rapeseed (http://apps.fas.usda.gov/psdonline/circulars/oilseeds.pdf). In China, a seedling transplant method is widely applied in rapeseed cultivation. Moreover, the “double-low” (low erucic acid and low glucosinolate) rapeseed seedling is also a popular vegetable, which can be eaten fresh or produced as a dried vegetable for export. A plant factory with LEDs may provide high precision and standardization of rapeseed seedlings for transplanting and vegetable cultivation. Rapeseed is sensitive to light quality (Li et al., 2013; Rondanini et al., 2014), and its responses to light quality should be investigated before the LEDs are applied in the plant factory. The present study set the R:B photonflux ratios from 100R:0B% to 0R:100B% with the same 550 μmolm^−2^s^−1^, which is close to the mean photonflux in the rapeseed field in lower reaches of Yangtze river on mid-September. Through a conjoint analysis of the morphological and photosynthetic characteristics of rapeseed leaves, the potential mutual links between them under R:B light quality were explained, and the basic mode of rapeseed leaf response to red and blue lights was summarized.

## Materials and methods

### Plant material and growth conditions

Seeds of *Brassica napus* L. cv. “Zhongshuang 11” were sown in a potted tray filled with organic nutrient soil (Peilei Organic Fertilizer Co., Zhenjiang, China). The tray was placed in a plant factory with cool white LEDs (Optrun CO., Nanjing, China) at 300 ± 15 μmolm^−2^s^−1^. The climate was set as follows: 22°*C*/18°*C* day/night temperature, a 12-h photoperiod and 80% relative humidity. Watering was performed with distilled water. One week after sowing, the seedlings with two cotyledons were transferred to big pots and then placed in LED-growth chambers (Optrun CO., Nanjing, China) for 3 weeks. The LED panels in the chambers mixed with red LEDs (peak wavelength 636 nm, full width at half maximum: 14 nm) and blue LEDs (peak wavelength 450 nm, full width at half maximum: 16 nm). The ratios of the R:B photonflux, including 100R:0B%, 75R:25B%, 50R:50B%, 25R:75B%, and 0R:100B% at 550 ± 20 μmolm^−2^s^−1^, were modulated by adjusting the operating current of the red and blue LEDs, respectively. The photonflux was measured by a quantum sensor (LI-COR, Lincoln, NE, USA).

### Leaf morphology measurements and microscopic observation for leaf cells

The leaf projected area (PA) and leaf surface area (SA) of the top second expanded leaves (TSLs) (*n* = 5) were scanned by a flat-bed scanner (Epson Expression 1680 1.0, Japan) and calculated by WinRHIZO (Regents Instruments, Quebec, Canada). The ratio of SA/PA was defined as the leaf wrinkle rate. The leaf midrib was cut out and scanned to measure the leaf tip angle, which was defined as the angle between the tangent line of the midpoint in the midrib and that of the tip point in the leaf. The leaf samples were dried and weighed for the LMA calculation.

A freehand cross-section was made for the leaf thickness measurement and the observation of palisade cells. Nail polish was painted on the surfaces of each leaf (*n* = 4); after solidification, the leaf was transferred to scotch tape for the leaf stoma observation. The leaf samples were immersed in 10% NaOH until they became transparent and then bleached in 10% NaClO for 2 h; after rinsing with water, the leaf samples were stained in 0.5% fast green dye for the observation of trichomes. The microscopic observation was performed under a fluorescent microscope (Leica DM2500, Leica Microsystems GmbH, Wetzlar, Germany). The measurements for the leaf cells were conducted using Image J software (http://rsb.info.nih.gov/ij/).

### Photosynthesis and the chlorophyll fluorescence measurement

The *P*_n_ of the TSLs (*n* = 5) was measured using a Li-6400 photosynthesis system (Li-Cor, Lincoln, NE, USA) with a clear top chamber (without light sources) in which the spectrum lights with 550 μmolm^−2^s^−1^ came from the LED-growth chambers. The photosynthesis irradiance response curve (*P*-*PPFD*) and CO_2_ response curve (*P*-*C*_i_) of the TSLs were measured by Li-6400 with a light source (Li-6400-02B LED, typical blue proportion: 13% at 100 μmolm^−2^s^−1^, 10% at 1000 μmolm^−2^s^−1^, and 7% at 2000 μmolm^−2^s^−1^). For *P*-*PPFD* curve measurement (*n* = 3) and the irradiance range, the CO_2_ concentration and temperature were controlled at 0–2200 μmolm^−2^s^−1^, 400 μmol CO_2_ m^−2^s^−1^ and 22 °C, respectively. For *P*-*C*_i_ curve measurement (*n* = 3), the saturation light was maintained at 1500 μmolm^−2^s^−1^, and the CO_2_ concentration was controlled according to a preset program at 400, 300, 200, 100, 50, 400, 600, 800, 1000, 1200, and 1400 μmol CO_2_ m^−2^s^−1^.

After a 12-h dark period, *F*_v_/*F*_m_ distribution in the TSLs (*n* = 3) and the top third expanded leaves (TTLs, *n* = 3) were measured by Imaging-PAM (Walz Gmbh, Effeltrich, Germany) and their mean value was calculated by ImagingWin software (Version 2.2). The smaller *F*_v_/*F*_m_ value indicated the more serious degree in PSII photodamage (Schreiber, 2004).

To analyze the response of the TSLs grown in 100R:0B%, 75R:25B%, 50R:50B%, 25R:75B%, and 0R:100B% to different BPF ratios, each selected leaf (*n* = 3) was flattened on a plastic board and was irradiated by LEDs at 550 μmolm^−2^s^−1^ in each treatment of the LED-growth chambers. The leaf-clips (2030-B, Walz Gmbh) were randomly attached on the leaf. After 20 min of dark adaption, the net *F*_v_/*F*_m_ was measured using a Mini-PAM (Walz Gmbh, Effeltrich, Germany). To measure the absolute *F*_v_/*F*_m_ (without PSII repair) of the TSLs (*n* = 3), the petiole of the selected leaves was incubated in 1 mM chloramphenicol, which can inhibit the synthesis of photosynthetic proteins and inhibit PSII repair (Nishiyama et al., 2001) under 30 μmolm^−2^s^−1^ white fluorescent lamps, and after 4 h, the absolute *F*_v_/*F*_m_ was measured using Mini-PAM.

To analyze the response of TSLs grown in the same light environment to different BPF ratios, the leaves under 50R:50B% were chosen and radiated by 100R:0B%, 75R:25B%, 50R:50B%, 25R:75B%, and 0R:100B% at 550 μmolm^−2^s^−1^; then, the *P*_n_ was rankly measured after 30 min adaptation, and the net and absolute *F*_v_/*F*_m_ were measured in parallel.

### Enzymatic determination for sucrose phosphate synthase (SPS) and sucrose synthase (SS)

The TSLs after 12 h of irradiation (*n* = 4) were used for the measurements. The methods of SPS and SS enzyme extraction were as described in Yu (1999) and Verma et al. (2011). The determination of SPS and SS were based on that of Verma et al. (2011).

### Reactive oxygen (ROS) determination and enzymatic determination for antioxidase

The samples from central part of the TSLs and TTLs after 12 h of irradiation (*n* = 4) were used to determine the reactive oxygen and antioxidase activities. The production rate of ${\text{O}}_{2}^{.-}$ was measured according to Able et al. (1998). The H_2_O_2_ content was measured according to Brennan and Frenkel (1977). The malondialdehyde (MDA) content was measured according to Kazemi et al. (2010). Enzymatic analysis of the superoxide dismutase (SOD), catalase (CAT), and peroxidase (POD) were measured according to the methods of Wang and Yang (2005), Aebi (1984) and Upadhyaya et al. (1985), respectively.

### Statistics analysis

The photosynthesis irradiance response curve was fitted to the exponential model of Bassman and Zwier (1991):

$$\begin{array}{l} P_{\text{n}}=P_{\text{max}}\left(1-e^{—\frac{\text{AQE }\times \;PPFD}{P_{\text{max}}}}\right)-R_{\text{d}} \end{array}$$

Where *P*_n_ is the net photosynthetic rate, *PPFD* is the photosynthetic photonflux density, AQE is apparent quantum yield, *P*_max_ is the photosynthesis capacity, and *R*_d_ is the dark respiration rate.

The CO_2_ response curve (*P*-*C*_i_) was fitted to the mode of Long and Bernacchi (2003):

$$\begin{array}{l} P_{\text{n}}=\text{min}\{\frac{V_{\text{cmax}}C_{\text{i}}}{C_{\text{i}}\;+\;K_{\text{c}}\left(1\;+\;O\;/\;K_{\text{o}}\right)},\frac{J_{\text{max}}C_{\text{i}}}{4.5{\text{C}}_{\text{i}}\;+\;10.5\Gamma^{*}},\\ \;\frac{3TPU}{\left(1\;-\;\Gamma^{*}\;/\;{\text{C}}_{i}\right)}\}\left(1\;-\;\frac{\Gamma^{*}}{C_{\text{i}}}\right)-{\text{R}}_{\text{d}} \end{array}$$

Where *C*_i_ is the intercellular CO_2_ concentration, *V*_cmax_ is the Rubisco maximum carboxylation rate, *J*_max_ is the maximum rate of electron transport, *TPU* is the rate of triose phosphate utilization, Γ^*^ is the photosynthetic compensation, *K*_c_ and *K*_o_ are the Rubisco Michaelis constant for CO_2_, and *O* is the concentration of oxygen in air.

Regression analysis, curve fitting and parameters calculation were performed with the SPSS software (Version 22.0, IBM, New York, USA). Pairwise multiple-comparison for data was carried out by one-way analysis of variance (ANOVA) in SPSS followed by Tukey's test (*P* = 0.05).

## Results

### Leaf morphology and anatomy

**Figure 1:**
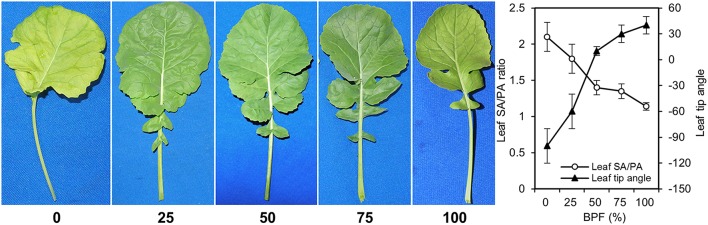
**The morphology of the top second expanded leaves of rapeseed grown under different R:B photonflux ratios**. The ratio of leaf surface area (SA) and leaf projected area (PA) reflects the wrinkle degree of leaves. For leaf tip angle, “−” denotes down-rolled, “+” denotes up-rolled. The BPF (%) denotes the proportion of blue light in the total photonflux. Mean ± Standard error; *n* = 5.

Under 100R:0B%, the wrinkles on the rapeseed leaf blade appeared significantly along with the veins, the SA/PA ratio was 2.1 times and the leaf margin rolled down with a −100° leaf tip angle (Figure 1). When the ratio of BPF increased to 100%, the SA/PA ratio was gradually reduced to 1.1, and leaf tip angle increased to 40° (Figure 1). The leaf lobe number in the leaf under 100R:0B% or 0R:100B% was only 1.2–2.0, whereas the number in the leaves under CLs was 3.2–3.6 (Table 1).

**Table 1:** **Different parameters of the top second leaves of rapeseed grown under R:B photonflux ratios**.

**Treatments**	**100R:0B%**	**75R:25B%**	**50R:50B%**	**25R:75B%**	**0R:100B%**
Leaf lobe number	1.2c	3.2a	3.6a	3.4a	2.0b
Leaf thickness (μm)	210d	296c	324b	344a	298c
AQE	0.026c	0.043a	0.035b	0.032b	0.022d
*P*max	9.8d	16.6b	18.2a	18.9a	10.8c
*J*max	112d	249a	205b	196c	78e
*V*cmax	21.5e	49.3a	39.0b	37.7c	23.1d
*TPU*	3.7d	9.0a	8.4b	8.2b	4.2c
*F*v/*F*m	0.787b	0.815a	0.812a	0.808a	0.783b
LMA (g/m^2^)	20.1e	38.3c	43.2b	51.9a	27.7d
Chlorophyll content (mg/g)	1.6d	2.7b	2.9a,b	3.1a	1.9c
Chl *a*:*b* (g/g)	2.4b	2.7a	2.8a	2.9a	2.8a
Carotenoid content (mg/g)	0.34d	0.68b	0.72b	0.81a	0.42c
Sucrose (mg/g)	4.7c	8.2a	7.0b	7.6a,b	7.0b
Starch (mg/g)	29.9c	39.2a	36.7b	34.8b	27.3d

*Letters behind the value indicate significant differences (Tukey's pairwise multiple-comparison test, P ≤ 0.05; n = 3–5)*.

**Figure 2:**
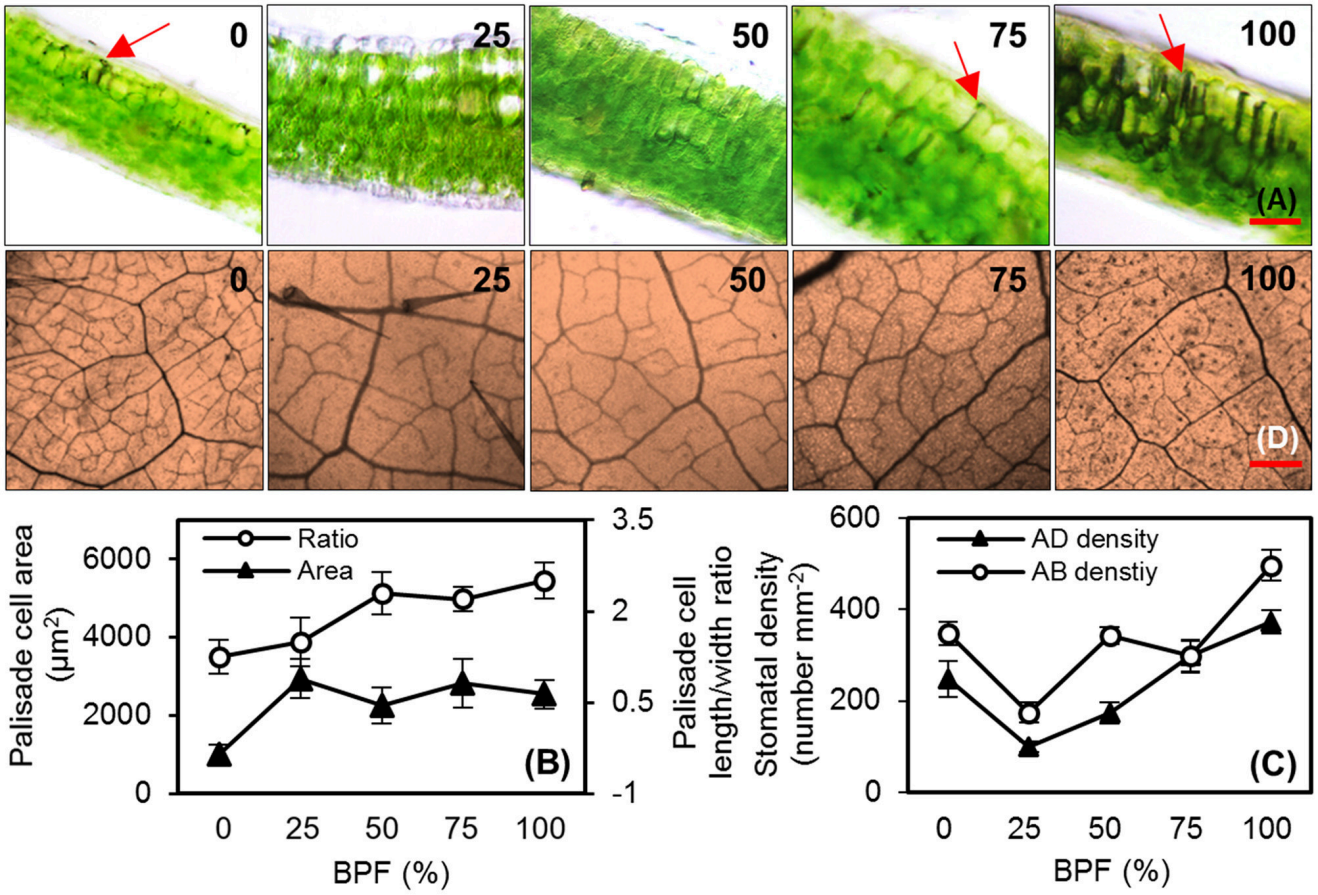
**The cell morphology of the top second expanded leaves of rapeseed grown under different R:B photonflux ratios**. **(A)** Micrographs of transverse sections of rapeseed leaves. The red arrows point to the black attachments between the palisade cells; scale bars = 100 μm. **(B)** The palisade cell area and length/width ratio observed in three random fields of view for each leaf slice. **(C)** The stomatal density of the abaxial (AD) and adaxial (AB) surfaces of leaf observed in three random fields of the solidified nail polish imprinted from leaf epidermis (not shown). **(D)** Leaf veins and trichomes viewed from the abaxial surface of rapeseed leaves; scale bars = 400 μm. The BPF (%) denotes the proportion of blue light in the total photonflux. Mean ± Standard error; *n* = 4.

100R:0B%-grown leaves were significantly thinner than the 0R:100B%-grown leaves. Under CLs, the leaf thickness increased with the increase of the BPF ratio (Table 1). 100R:0B%-grown leaves had only one layer of palisade cells, whereas 0R:100B%- and CLs-grown leaves had two layers (Figure 2A, Table 1). The shape of the 100R:0B%-grown palisade cells was nearly spherical with a low length/width ratio (1.0–1.5) from a cross-sectional observation. When the BPF ratio increased to 100%, the palisade cells gradually elongated with an increased length/width ratio (2.0–3.0) (Figures 2A,B). The 100R:0B%-grown palisade cells were smaller compared to the 0R:100B%- and CLs-grown cells (Figure 2B). Many black attachments that were enriched in the intercellular space were observed in the 100R:0B%- and 0R:100B%-grown leaves, whereas 25R:75B%-grown leaves showed relatively little black attachment (Figure 2A). The stomatal density of the abaxial and adaxial sides of 100R:0B%-grown leaves was lower than that of the 0R:100B%-grown leaves. Under the CLs, the stomatal density of the abaxial and adaxial sides increased with the increase of the BPF ratio (Figure 2C). A large number of epidermal trichomes along with the main vein and the small veins (Figure 2D, density at 0.6 ± 0.2 per mm^2^) were observed on the abaxial surface of 75R:25B%-grown leaves.

### Leaf photosynthesis

**Figure 3:**
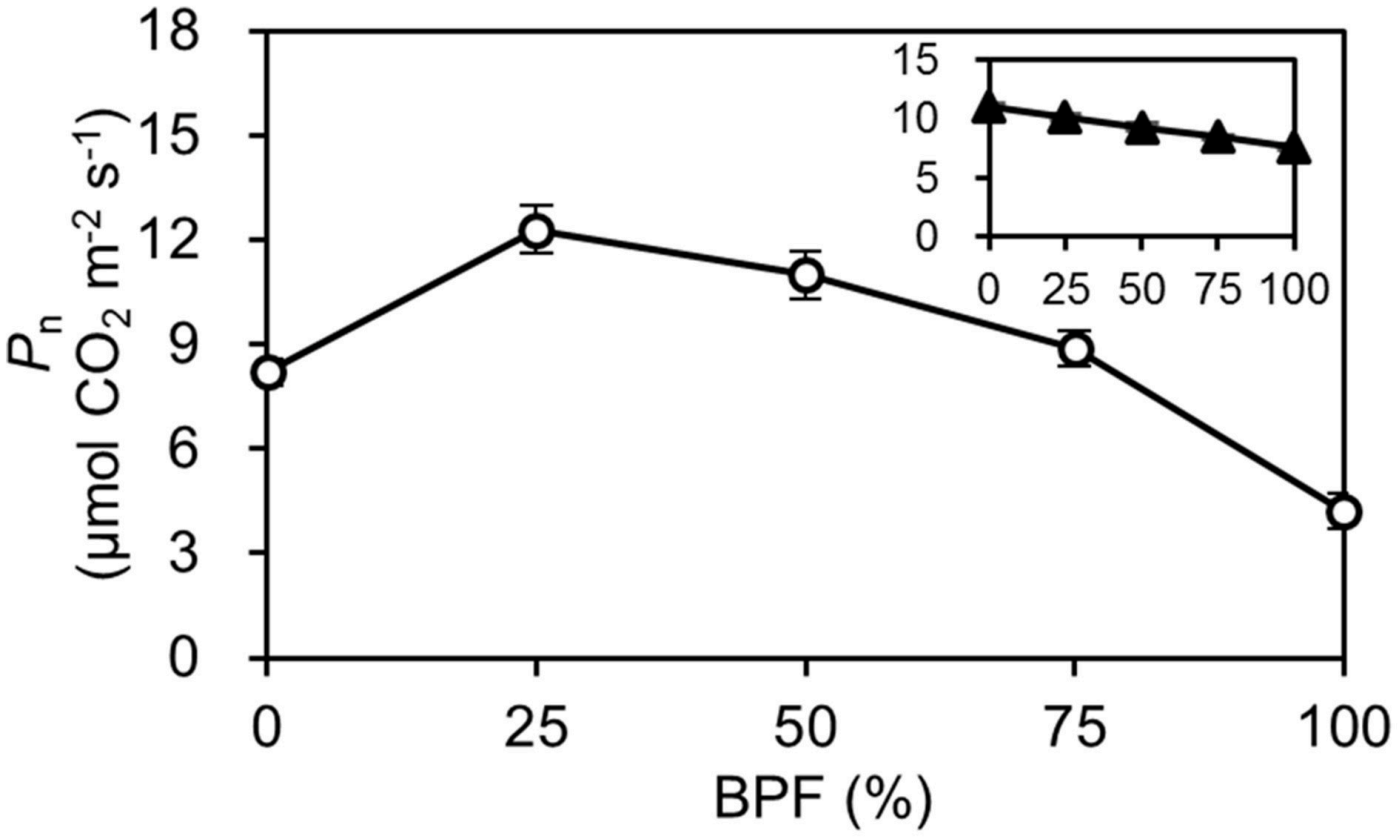
**The net photosynthetic rate (***P***_**n**_) of the top second expanded leaves of rapeseed under different R:B photonflux ratios**. The line with open symbols denotes the *P*_n_ of the rapeseed leaves grown under different R:B photonflux ratios. The actinic light spectrum was identical to that during growth. The line with triangle symbols in the mini-graph denotes the *P*_n_ of the rapeseed leaves grown in the same light environment (50R:50B%) to different R:B photonflux ratios. The BPF (%) denotes the proportion of blue light in the total photonflux. Mean ± Standard error; *n* = 5.

**Figure 4:**
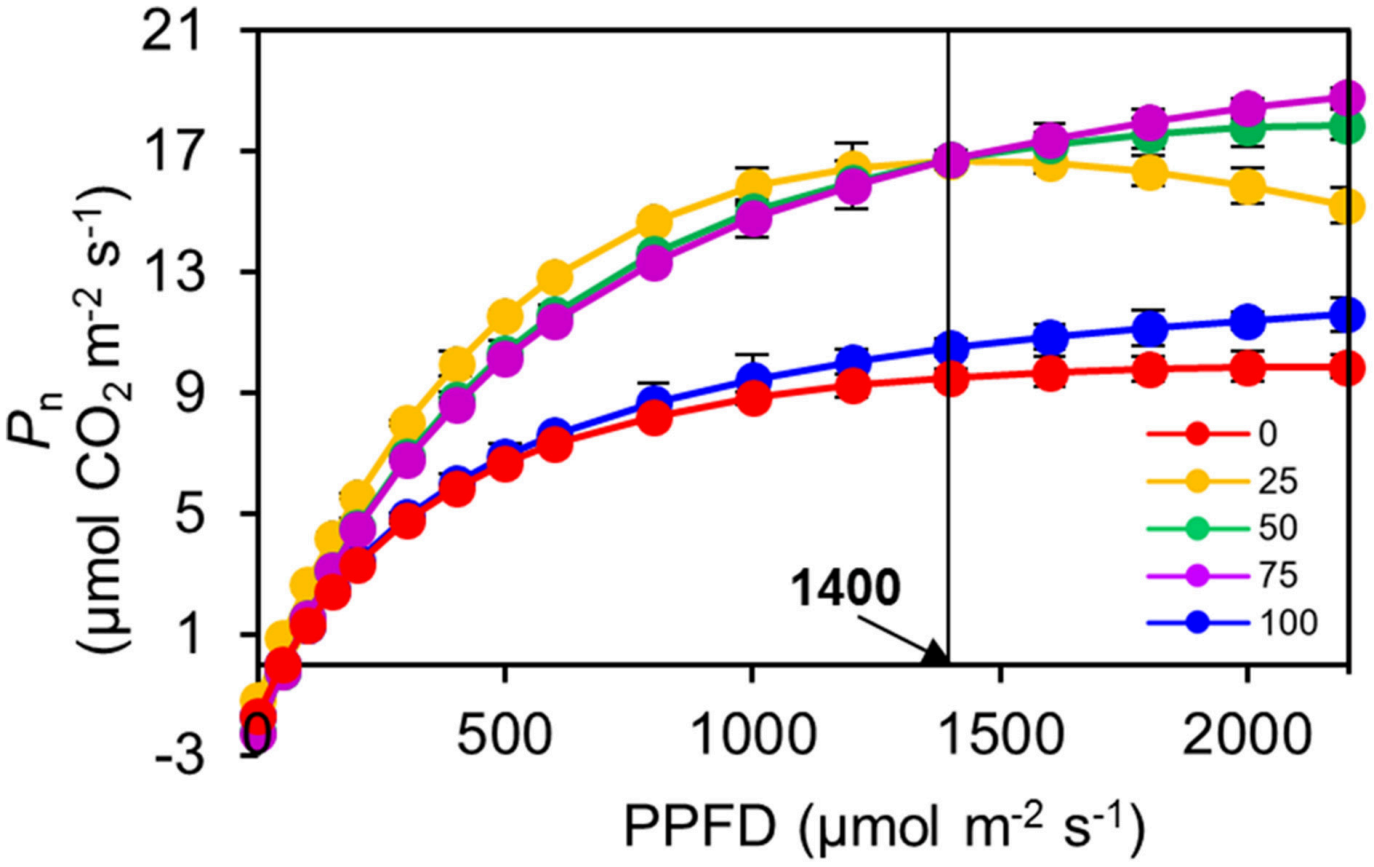
**Response of net photosynthetic rate (***P***_**n**_) to irradiance (***PPFD***) for the top second expanded leaves of rapeseed grown under different R:B photonflux ratios determined by standard Li-6400-02B light source**. The light saturation of the 75R:25B%-grown leaves was approximately 1400 μmolm^−2^s^−1^, whereas other treatments did not show obvious light saturation until 2200 μmolm^−2^s^−1^. Mean ± Standard error; *n* = 3.

The *P*_n_ of the 100R:0B%-grown leaves was 49.5% higher than that of the 0R:100B%-grown leaves. Under CLs, the *P*_n_ gradually decreased with the increase of the BPF ratio (Figure 3). The *P*-*PPFD* was measured using Li-6400-02B light and showed that the light saturation of the 75R:25B%-grown leaves was approximately 1400 μmolm^−2^s^−1^, whereas other treatments did not show obvious light saturation until 2200 μmolm^−2^s^−1^ (Figure 4). The AQE of the 100R:0B%-grown leaves was significantly higher than that of the 0R:100B%-grown leaves, and the AQE of the 75R:25B%-grown leaves was significantly higher than those of 50R:50B%- and 25R:75B%-grown leaves (Table 1). The trend of *P*_max_ was different with AQE. The *P*_max_ of the 100R:0B%-grown leaves was significantly lower than that of the 0R:100B%-grown leaves, and the *P*_max_ of the 75R:25B%-grown leaves was significantly lower than that of the 50R:50B%- and 25R:75B%-grown leaves. Furthermore, the AQE and *P*_max_ of the 100R:0B%- and 0R:100B%-grown leaves were significantly lower than those of the CLs-grown leaves. The *J*_max_ of the 100R:0B%-grown leaves was higher than that of 0R:100B%-grown leaves, and the trend of the *V*_cmax_ and *TPU* was opposite of the *J*_max_. The *J*_max_, *V*_cmax_ and *TPU* under CLs decreased with an increase of the BPF ratio and were significantly higher than those under 100R:0B% and 0R:100B% (Table 1).

### LMA, pigment, carbohydrate content, and enzyme activity of SPS and SS

**Figure 5:**
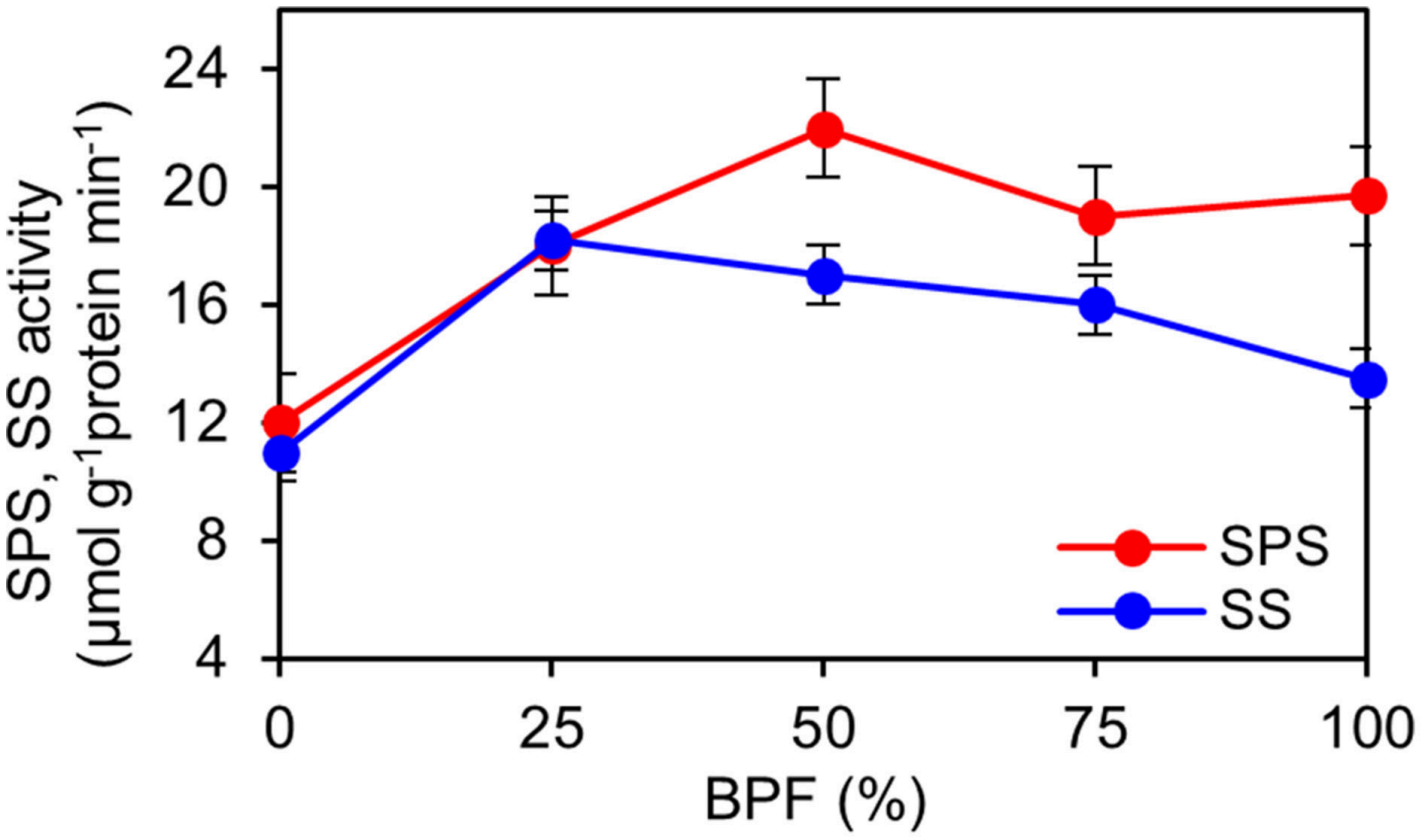
**Effect of different R:B photonflux ratios on sucrose phosphate synthase (SPS) and sucrose synthase (SS) activities in the top second expanded leaves of rapeseed**. The BPF (%) denotes the proportion of blue light in the total photonflux. Mean ± Standard error; *n* = 4.

The indexes of LMA, Chl and carotenoid content per unit leaf weight under 100R:0B% were significantly lower than those under 0R:100B%, and these indexes under the CLs increased with the increase of the BPF ratio and were significantly higher than those under 100R:0B% and 0R:100B% (Table 1). The Chl *a*:*b* of the 100R:0B%-grown leaves was only 2.4, which was significantly lower than those of the 0R:100B%- and CLs-grown leaves (approximately 2.8). The sucrose content of the 100R:0B%-grown leaves was at least 32.9% lower than that of the 0R:100B%- and CLs-grown leaves. The sucrose content of the 0R:100B%-grown leaves was not significantly different compared with that of the 50R:50B%- and 25R:75B%-grown leaves, but was 14.6% lower than that of 75R:25B%-grown leaves (Table 1). The starch content of 100R:0B%-grown leaves was significantly higher than that of the 0R:100B%-grown leaves. Under the CLs, the starch content decreased with the increase of the BPF ratio (Table 1). The SPS activity of the 0R:100B%-grown leaves was not significantly different compared with that of CLs-grown leaves and was significantly higher than that in the 100R:0B%-grown leaves. The SS activity in the 100R:0B%-grown leaves was lower than that in the 0R:100B%-grown leaves. Under the CLs, the SS activity gradually decreased with the increase of the BPF ratio (Figure 5).

### *F*_v_/*F*_m_, ROS, and antioxidase activity

*F*_v_/*F*_m_ of the TSLs all showed a homogenous distribution (similar with **Figure 7A**). The mean values of *F*_v_/*F*_m_ in the 100R:0B%- and 0R:100B%-grown leaves were between 0.78 and 0.79, which showed a stress characteristic, whereas the value was > 0.80 in the CLs-grown leaves, which was similar to a healthy leaf (Table 1).

**Figure 6:**
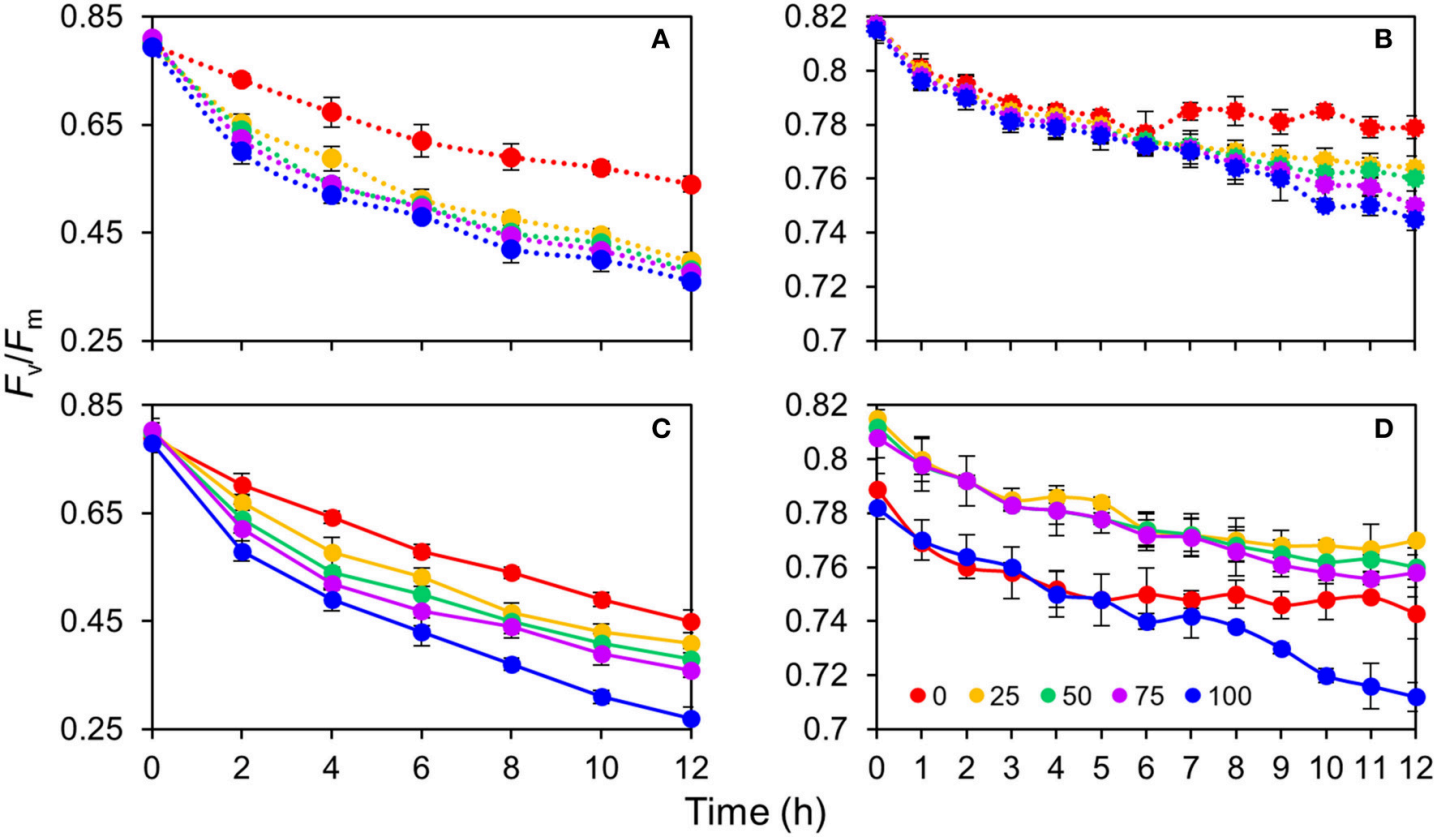
**The change of photosynthetic maximum quantum yield (***F***_**v**_/***F***_**m**_) for the top second expanded leaves of rapeseed under different R:B photonflux ratios in 12 h of irradiation**. **(A,B)** The absolute and net *F*_v_/*F*_m_ change of the leaves grown in the same light environment (50R:50B%) to different BPF ratios. **(C,D)** The absolute and net *F*_v_/*F*_m_ change of the leaves grown in 100R:0B%, 75R:25B%, 50R:50B%, 25R:75B%, and 0R:100B% to the different BPF ratios. The leaves measured for the absolute *F*_v_/*F*_m_ change **(A,C)** were pre-treated with 1 mM chloramphenicol for 4 h, whereas the leaves measured for the net *F*_v_/*F*_m_ change **(B,D)** were not pre-treated with chloramphenicol. The BPF (%) denotes the proportion of blue light in the total photonflux. Mean ± Standard error; *n* = 3.

The response of the leaves grown in the same light environment (50R:50B%) to different BPF ratios is shown in Figures 6A,B. The absolute *F*_v_/*F*_m_ declined gradually with time and its rate of decline was lowest under 100R:0B% (approximately 0.02 per hour) and abruptly increased under B-containing spectra (approximately 0.03–0.04 per hour). A greater blue light proportion induced a more rapid decline of the absolute *F*_v_/*F*_m__._ The declining rate of net *F*_v_/*F*_m_ was significantly lower than that of the absolute *F*_v_/*F*_m_. When irradiated for 6 h under 100R:0B% and for 9 h of irradiation under 75R:25B% and 50R:50B%, a plateau of the net *F*_v_/*F*_m_ appeared but was absent under 25R:75B% and 0R:100B%.

The response of the TSLs grown in 100R:0B%, 75R:25B%, 50R:50B%, 25R:75B%, and 0R:100B% to different BPF ratios is shown in Figures 6C,D. A greater blue light proportion induced a more rapid decline of the absolute *F*_v_/*F*_m_, but the *F*_v_/*F*_m_ advantage of 100R:0B%-grown leaves was not so remarkable. The declining rates of the net *F*_v_/*F*_m_ in the 100R:0B%- and 0R:100B%-grown leaves were more rapid than that in the CLs-grown leaves.

**Figure 7:**
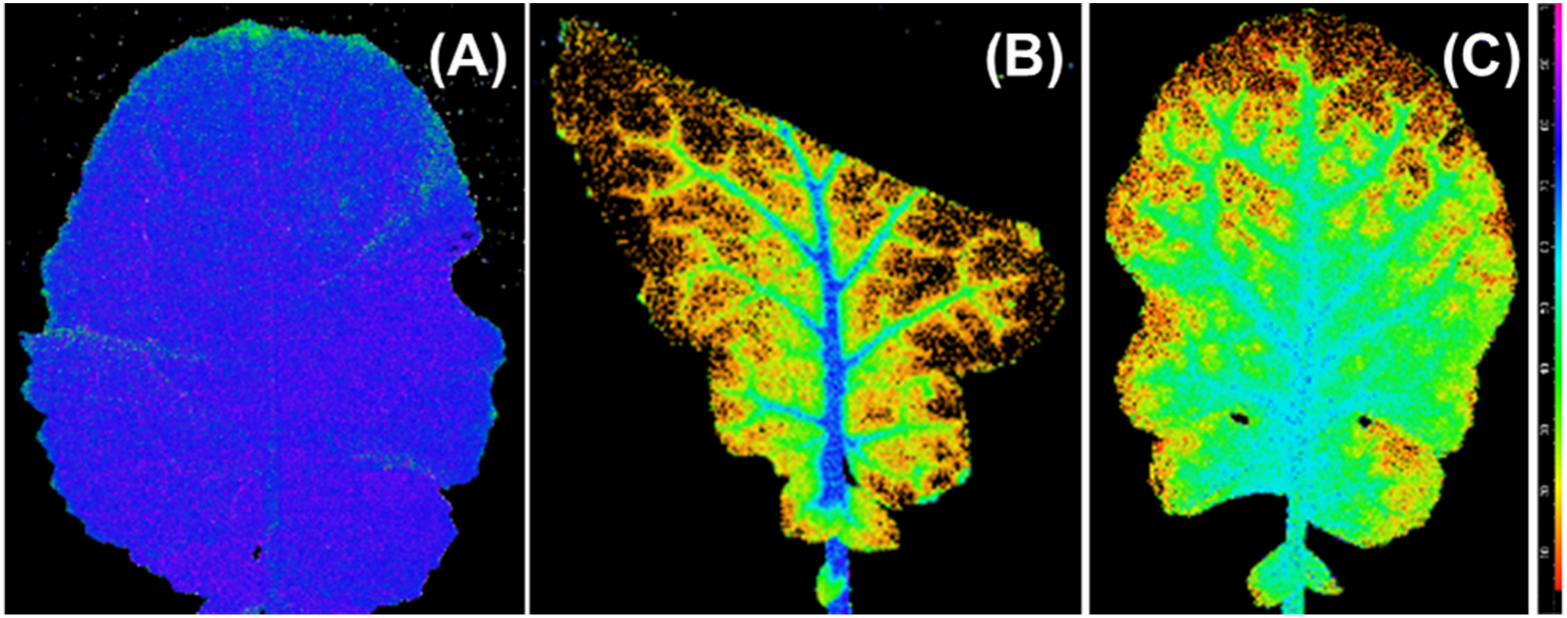
**An image of the distribution of photosynthetic maximum quantum yield (***F***_**v**_/***F***_**m**_) over the top third leaves grown under 50R:50B% (A), 100R:0B% (B), and 0R:100B% (C)**. The images of the top third leaves grown under 75R:25B% and 25R:75B% lights are similar with leaves grown under 50R:50B%. The *F*_v_/*F*_m_ scale (× 100) is shown at the right.

The *F*_v_/*F*_m_ distribution of the 100R:0B%- and 0R:100B%-grown TTLs showed a heterogeneous distribution (mean value < 0.5), and the *F*_v_/*F*_m_ value in the regions adjacent to the veins was conspicuously higher than that in other regions (Figures 7B,C). In contrast, the *F*_v_/*F*_m_ distribution in the CLs-grown leaves was homogeneous with a mean *F*_v_/*F*_m_ = 0.8.

**Figure 8:**
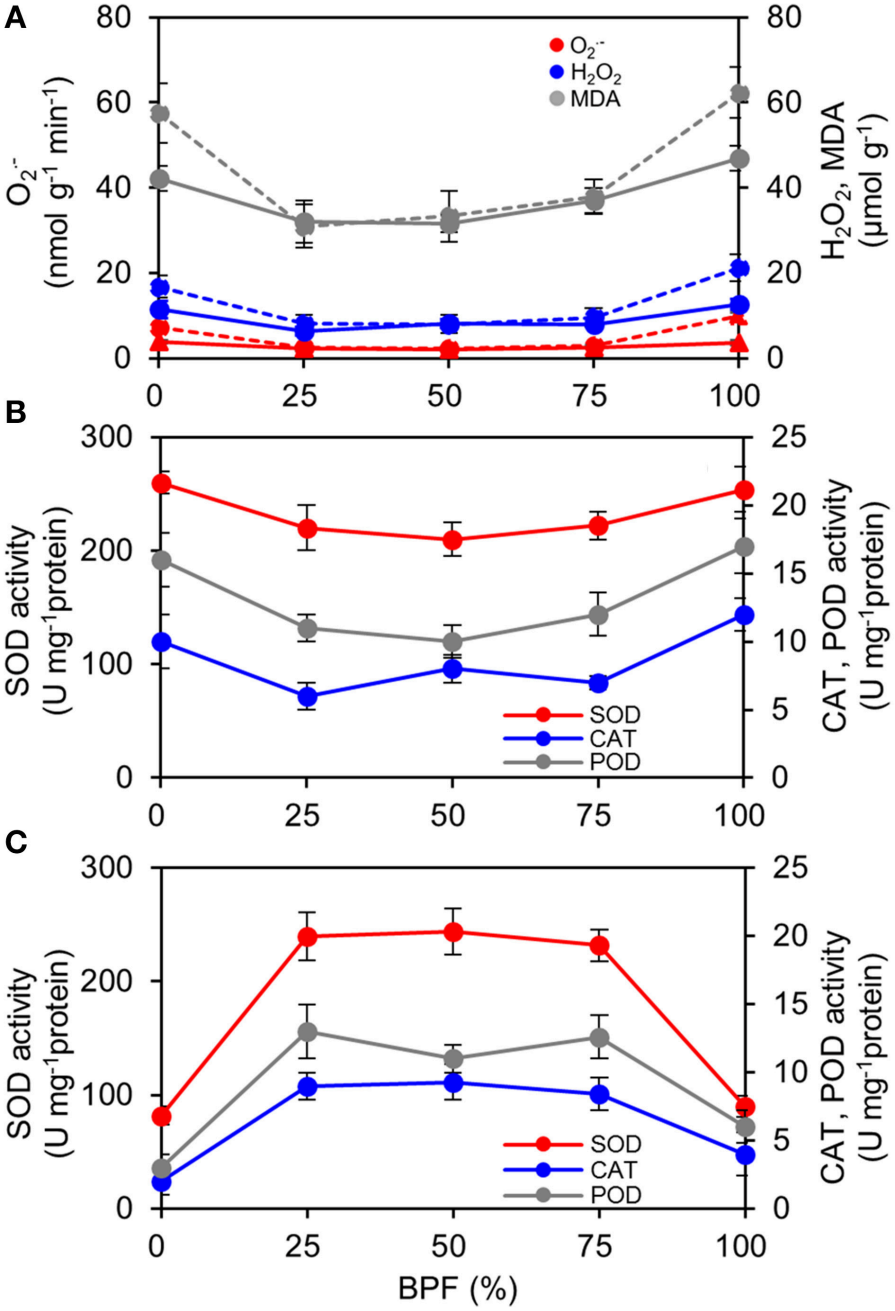
**Effect of different R:B photonflux ratios on the content of reactive oxygen species and the antioxidase activities in the top second and top third leaves (TSLs and TTLs) of rapeseed**. **(A)** The production rate of ${\text{O}}_{2}^{.-}$, and H_2_O_2_and MDA content. The solid lines indicate the parameters of TSLs and the dash lines indicate the parameters of TTLs. **(B)** Activities of SOD, CAT, and POD in TSLs. **(C)** Activities of SOD, CAT, and POD in TTLs. The BPF (%) denotes the proportion of blue light in the total photonflux. Mean ± Standard error; *n* = 4.

The content of ROS (${\text{O}}_{2}^{.-}$ and H_2_O_2_) of the 100R:0B%- and 0R:100B%-grown TSLs was significantly higher than that of CLs-grown TSLs (Figure 8A). Correspondingly, the ${\text{O}}_{2}^{.-}$ removal ability (SOD activity) and H_2_O_2_ scavenging ability (CAT and POD activity) of the 100R:0B%- and 0R:100B%-grown TSLs were higher than those of the CLs-grown TSLs (Figure 8B). The ROS content of the TTLs showed a similar trend with the TSLs. Grown under CLs, the ROS content showed no difference between the TTLs and TSLs. Grown under 100R:0B% and 0R:100B%, the ROS content of the TTLs was significantly higher than that of the TSLs (Figure 8A). The ROS scavenging ability of the 100R:0B%- and 0R:100B%-grown TTLs was remarkable weak compared to the CLs-grown TTLs and 100R:0B%- and 0R:100B%-grown TSLs (Figures 8B,C). Moreover, the peroxidation level of the lipid (MDA content) synchronously changed with the ROS content (Figure 8A).

## Discussion

### Leaf morphogenesis response to different R:B photonflux ratios

The shapes of rapeseed leaves, which were wrinkled blade and down-rolled margin grown under 100R:0B% and 75R:25B% (Figure 1), were similar to the appearance of the “shade leaf” in nature that was grown under a shade condition with low light intensity, a low proportion of R and a high proportion of FR (reduced R:FR ratio) and a high proportion of green (G) (Casal, 2013). However, the shapes of rapeseed leaves, which were flat blade and slightly up-rolled margin grown under 0R:100B% and 25R:75B% (Figure 1), were similar to the “sun leaf” that was grown under a no-shading condition. Along with the change of the R:B photonflux ratio from 100R:0B% to 0R:100B%, the change of the leaf shape looks similar to the shade removing process of leaf. At the anatomical level, the monolayer of palisade cells of 100R:0B%-grown leaves (Figure 2) is also consistent with the classical “shade leaf” (Yano and Terashima, 2001). Macedo et al. (2011) found that the boundary of the palisade and spongy mesophyll tissues of the *Alternanthera brasiliana* leaves grown under 100R:0B% was not clear, which is consistent with the leaves under dark conditions. Schuerger et al. (1997) found that the anatomical features of the pepper leaves were similar among the plants grown under 100R% or 83R:17FR%. When the BPF ratio increased above 25% (and possibly lower), the normal two layers of the cells in the palisade tissue appeared (Figure 2), which indicated the decisive role of blue light for the development of the palisade tissues. The quantitative increase in the length/width ratio of the palisade cells with the increasing BPF ratio reflected the deepening of the compaction degree of the palisade tissue. Lewis (1972) reported that the palisade mesophyll of the “sun leaf” was more compact than the “shade leaf.” The isolated finding regarding the high density of trichomes on the abaxial surface of the 75R:25B%-grown leaves (Figure 2) maybe a shade characteristic. Macedo et al. (2011) found that the number of trichomes on both surfaces of the *A. brasiliana* leaf increased in the dark conditions compared to light conditions.

The thickness of the epidermis, palisade and spongy tissues grown under 0R:100B% was higher than that grown under 100R:0B% (Macedo et al., 2011). Schuerger et al. (1997) found that the leaf thickness, numbers of chloroplasts per palisade mesophyll cell, and thickness of the palisade and spongy mesophyll tissues under the blue-containing spectra were higher than under the 100R% and 83R:17FR%. We observed that the blue light enhanced the leaf thickness and promoted the LMA, stomatal density, chlorophyll content per weight and Chl *a*:*b* of rapeseed leaves (Table 1). Hogewoning et al. (2010) found that the LMA, stomatal density and Chl content per area of the cucumber leaf showed an increasing dose along with the R:B photonflux ratio change from 85R:15B% to 50R:50B%. Under the natural condition, the leaf thickness, LMA, chlorophyll content and Chl *a*:*b* increase with the irradiation intensity enrichment (Björkman, 1981; Terashima and Hikosaka, 1995). Hogewoning et al. (2010) indicated that the dose change of several leaf morphology indexes of cucumber with an increase of BPF have a comparable relationship as reported for leaf responses to irradiance intensity, and our findings confirmed his viewpoint. It may be concluded that blue light is conducive to the sun-type morphogenesis for leaves.

A high photosynthetic rate in nature is closely related to the sun-type morphogenesis of the leaf, especially leaf thickening and vice versa (Terashima et al., 2001, 2006). We found that 100R:0B% induced a higher *P*_n_ than 0R:100B%, and CLs with a greater R-ratio induced a higher *P*_n_; however, the morphogenesis of leaves grown in the 100R:0B% or CLs with a greater R-ratio did not show the sun-type characteristics and vice versa (Figure 3). Casal (2013) suggested that photoreceptors, especially phytochrome and cryptochrome, were very important for morphogenesis. The R-excited phytochrome Pfr form and B-excited cryptochrome are two resistant agents for the shade symptom induced by far-red light, green light, or the dark treatment. Under the R:FR combinations, the far red light is able to induce shade symptoms through triggering of the accumulation of the phytochrome Pr form with biological inactivity, whereas red light can resist this effect through exciting phytochrome to the Pfr form with biologically activity (Casal, 2013). Under the B:G combinations, blue light is considered the main effective component, which can gradually restrain the “shade syndrome” with a dose increase, and cryptochrome plays a key role in this process (Sellaro et al., 2010). However, when red and blue lights were applied alone in the present study, the leaves grown under the high red-photonflux (RPF) ratio also were prone to show shade-type characteristics; these findings indicate that Pfr and cryptochrome have different shade inhibiting ability for the leaves and the effect of Pfr is obviously weaker than the cryptochrome. However, this phenomenon is only limited in the leaf. The study for the stem/hypocotyl responses of eggplant (Hirai et al., 2006), petunia (Fukuda et al., 2016), and cucumber (Hernández and Kubota, 2016) to different R:B photonflux ratios showed that the length of the stem/hypocotyl grown under 0R:100B% was much higher than the R-containing lights. As we know, the elongation of the stem/hypocotyl also belongs to the “shade syndrome.”

The shade- or sun-type morphogenesis of leaves induced by red or blue light may be independent of the light intensity under a low or moderate irradiance. The investigation of the 100R:0B%-grown leaves of joyweed under only 20 μmolm^−2^ s^−1^ (Macedo et al., 2011), cucumber under 100 μmolm^−2^ s^−1^ (Hogewoning et al., 2010; Hernández and Kubota, 2016), pepper under 330 μmolm^−2^ s^−1^ (Schuerger et al., 1997), and rapeseed in the present study under 550 μmolm^−2^ s^−1^ showed shade-type characteristics compared with the 0R:100B%-grown leaves. However, the discussion did not contain the studies for tissue cultural plants, the results of which were very specific because of a cross effect of the addition of hormones (such as 2,4-D, 6-BA, and NAA) in the medium (Li et al., 2010, 2013).

### Photosynthetic carbon accumulation response to different R:B photonflux ratios

For the plants grown under an artificial environment and provided constant light quality and intensity, *P*_n_ is the most important index for elucidating the photosynthetic ability. We observed that the red light was more conducive to improve *P*_n_ compared to the blue light (Figure 3). Sabzalian et al. (2014) also found that the *P*_n_ of *Mentha* plants grown under 100R:0B% was higher than under 0R:100B% (500 μmolm^−2^ s^−1^). However, Hogewoning et al. (2010) and Hernández and Kubota (2016) found a different trend for cucumber leaf, which indicated that the leaf *P*_n_ increased with the increase of the BPF ratio under 100 μmolm^−2^ s^−1^. Under different light qualities, leaf photosynthesis depends on the spectrum preference of the photosynthetic apparatus and the effect of the leaf morphogenesis. The measurement of the rapeseed leaves with the same background showed that the photosynthetic apparatus is prone to accept red light (Figure 3), and Loreto et al. (2009) found the same result in cucumber leaves. This can be attributed to the classical McCree relative quantum efficiency curve (McCree, 1972). For decades, the explanation for this phenomenon has gradually been refined and can be summarized as follows: (1) red light has a lower non-effective absorption by carotenoids and non-photosynthetic components compared with blue light, which correspond to a highly effective light energy absorption by chlorophyll for photosynthesis (Evans, 1987); (2) red light enhances leaf mesophyll conductance compared with blue light (Loreto et al., 2009); and 3. red light is healthier than blue light for PSII (only 64% of damage rate in contrast with the blue light over 12 h, Figure 6A). In the short-term, photosynthesis is greatly independent from the effect of leaf morphogenesis. Although blue light can promote the stomatal opening through activating phototropin (Inoue et al., 2010), Loreto et al. (2009) reported that the stomatal conductance showed no significant change under different R:B photonflux ratios.

We found that the leaves grown under 0R:100B% or CLs with a high BPF ratio showed a higher ability (higher *P*_max_) to utilize high photonflux, and the leaves grown under 100R:0B% or CLs with low BPF ratios showed higher efficiency (AQE) in utilizing low photonflux. The obvious performance of this phenomenon was that the *P*_n_ of the leaves under 75R:25B% treatment was higher than that under of 50R:50B% and 25R:75B% when photonflux was below 1400 μmolm^−2^ s^−1^, and when photonflux was above 1400 μmolm^−2^ s^−1^, the *P*_n_ of the leaves under 75R:25B% treatment was lower than that under 50R:50B% and 25R:75B% (Figure 4). Different light spectra caused significant differences in leaf morphogenesis, which may greatly effect leaf photosynthesis (Figure 4). The morphogenesis of leaves grown under a high BPF ratio benefited photosynthetic capacity, and this may be explained as follows: (1) the flat and slightly up-rolled leaf can receive more light energy compared with a wrinkled and down-rolled leaf; (2) the leaves with high LMA and thickness and compacted palisade tissue have a large photosynthetic tissue volume (Boardman, 1977); (3) the leaves with high stomatal density promote stomatal conductance (Boardman, 1977); (4) the leaves with a sun-type chloroplast structure, which is also induced by blue light, is associated with a high photosynthetic ability (Lichtenthaler et al., 1980); and (5) the leaves with more Chl content are also a benefit for photosynthesis.

The cucumber leaves grown under 100 μmolm^−2^ s^−1^ appeared to have a high *P*_n_ and an increasing BPF ratio (Hogewoning et al., 2010, Hernández and Kubota, 2016). However, under 500 μmolm^−2^ s^−1^ and compared to 0R:100B%, the 100R:0B% resulted in a higher *P*_n_ for the *Mentha* plants (Sabzalian et al., 2014), and the same result was observed in rapeseed under 550 μmolm^−2^ s^−1^. We also found that the *P*_n_ of leaves under CLs increased with an increase in the RPF ratio. We conjectured that red and blue lights may play mutual roles in *P*_n_ for an extended time of irradiation. The mutual roles of red and blue lights may be affected by the differences in the species and irradiation intensity (among others). When the red light dominated, the leaf *P*_n_ showed an R-preference. When the blue light dominated, the *P*_n_ showed a B-preference.

Sucrose and starch are two important products of leaf photosynthesis. SPS plays an import role in the regulation of the balance of sucrose synthesis and degradation (Huber and Huber, 1996). Synchronously higher SPS activity and sucrose content in B-containing treatments compared to that in the 100R:0B% treatment (Figure 5, Table 1) implied an essential role of blue light for sucrose synthesis. The 100R:0B% and CLs with a high RBF ratio induced higher starch content than 0R:100B% (Table 1), which indicates that red light is benefit for starch accumulation. Sæbø et al. (1995) suggested that red light may inhibit the translocation of photosynthates out of the leaves.

### Monochromatic red or blue light stress

Hogewoning et al. (2010) indicated that 100R:0B% was an adverse spectrum for plant growth and induced physiology disorder, which was defined as “red light syndrome.” This syndrome was described as impaired photosynthesis including a low *F*_v_/*F*_m_, unresponsive stomatal conductance and a low *P*_max_. Similar symptoms of low *F*_v_/*F*_m_, AQE and *P*_max_ were observed in the 0R:100B%-grown rapeseed leaves under 550 μmolm^−2^s^−1^ (Table 1) and this may be described as “blue light syndrome.” However, “blue light syndrome” did not appear in potatoes (Kim and Lee, 2004), lettuce (Johkan et al., 2010), and cucumbers (Hogewoning et al., 2010) under 0R:100B% with a low photonflux (=200 μmolm^−2^s^−1^). We inferred that the 0R:100B% with a high photonflux may induce “blue light syndrome.”

Compared with PSI, PSII is extremely sensitive to light stress and is commonly damaged prior to PSI (Sonoike, 2011). The decreased degree of the *F*_v_/*F*_m_ (Figure 6) indicated the degree of PSII photodamage. In addition, the BPF ratio and the absolute PSII photodamage were increased (Figures 6A,C). The PSII photodamage gap between the 100R:0B% and B-containing treatments (Figure 6A) showed an acceleration effect of PSII photodamage induced by the blue light. According to a two-step photodamage mode of PSII by Ohnishi et al. (2005), blue light first induces the inactivation of the oxygen-evolving complex and then blue or other spectra are able to inhibit the activity of the PSII reaction center. When the repair rate was lower than the photodamage rate, the *F*_v_/*F*_m_ declined and net PSII photodamage occurred; however, when the repair and photodamage rate were in a balance, the *F*_v_/*F*_m_ was maintained on a plateau and the net PSII photodamage did not occur (Figures 6B,D). The 50R:50B%-grown leaves with net PSII photodamage had a similar repair ability under 100R:0B%, 0R:100B%, and CLs (Figures 6A,B). Grown under 100R:0B%, 0R:100B%, and CLs, the trend of net PSII photodamage deviated from the trend of the absolute PSII photodamage (Figures 6A,C), and the degree of net PSII photodamage of the leaves grown under 100R:0B% and 0R:100B% was higher than that under CLs, which indicated that the low PSII repair ability of leaves under 100R:0B% and 0R:100B% resulted in PSII photodamage.

In 100R:0B%- and 0R:100B%-grown leaves, we observed a higher ROS level, damaged cellular membrane structure (Figure 8) and rich black attachments among the intercellular space and cell wall (Figure 2). The formation of ROS and peroxidases can initially cross-link phenolic compounds and glycoproteins of the cell walls causing it to stiffen (Tenhaken, 2015). It was inferred that ROS induced the rich black attachments.

Hogewoning et al. (2010) found that only 7% blue light was sufficient to prevent any overt dysfunctional photosynthesis and suggested that the exciting phytochrome induced “red light syndrome” because the cryptochrome and phototropin were not excited under 100R:0B%. Trouwborst et al. (2016) found that the leaves injured by 100R:0B% could recover from photodamage within 4 days after switching from 100R:0B% to 70R:30B% and that *P*_n_ at a growth irradiance could increase to the same level as in healthy leaves. Hoffmann et al. (2015) even found high blue light can enhance the secondary metabolism to strengthen the repair ability of leaves to UV damage. We observed that the red light prevented dysfunctional photosynthesis induced by the blue light. The relative amount of phytochrome in 0R:100B%-grown cucumber leaves was lower than that in 100R:0B%- and CLs-grown leaves (Hogewoning et al., 2010, 2012). We suggest that “blue light syndrome” may be induced by the lack of active phytochrome.

## Conclusion

Blue light is conducive to the sun-type morphogenesis of rapeseed leaves, which showed a sun-type leaf phenotype and anatomical structure when irradiated with 0R:100B% or CLs with a high BPF ratio. The leaves grown under 0R:100B% or CLs with a high BPF ratio showed a higher ability to utilize high photonflux. When the blue light dominated, the *P*_n_ showed a B-preference. Red light is beneficial to the photosynthetic apparatus. The leaves grown under 100R:0B% or CLs with a low BPF ratio showed higher efficiency in utilizing low photonflux. When the red light dominated, the *P*_n_ showed an R-preference. The 100R:0B%-grown rapeseed leaves under 550 μmolm^−2^s^−1^ showed classical “red light syndrome,” while the 0R:100B%-grown leaves showed “blue light syndrome.” The CLs were suitable for *P*_n_ and the growth of rapeseed seedlings.

## Author contributions

CS performed the experiments, analyzed the data, wrote and revised the manuscript. LC, YX, and CS helped in conducting the experiments. JX and LX helped in analyzing the data. XZ and GR designed the research and critically edited the manuscript. All authors approved the final manuscript.

### Conflict of interest statement

The authors declare that the research was conducted in the absence of any commercial or financial relationships that could be construed as a potential conflict of interest.

## Abbreviations

LEDs: light-emitting diodes
RPF: red-photonflux
BPF: blue-photonflux; compound lights (CLs)
FR: far-red light
G: green light
*F*_v_/*F*_m_: maximum quantum yield in the dark
Chl: chlorophyll
PS: photosystem
TSLs: top second expanded leaves
TTLs: the top third expanded leaves
PA: projected area
SA: surface area
LMA: leaf mass per area
*P*_n_: net photosynthetic rate
AQE: apparent quantum yield
*P*_max_: photosynthesis capacity
*J*_max_: maximum rate of electron transport
*V*_cmax_: Rubisco maximum carboxylation rate
*TPU*: rate of triose phosphate utilization
SPS: sucrose phosphate synthase
SS: sucrose synthase
ROS: reactive oxygen species
SOD: superoxide dismutase
CAT: catalase
POD: peroxidase
MDA: malondialdehyde.

## References

[B1] Able A. J. Guest D. I. Sutherland M. W. (1998). Use of a new tetrazolium-based assay to study the production of superoxide radicals by tobacco cell cultures challenged with avirulent zoospores of *Phytophthora parasitica* var nicotianae. Plant Physiol. 117, 491–499. 10.1104/pp.117.2.491 9625702 PMC34969

[B2] Aebi H. (1984). Catalase *in vitro*. Methods Enzymol. 105, 121–126. 10.1016/S0076-6879(84)05016-3 6727660

[B3] Bassman J. H. Zwier J. C. (1991). Gas exchange characteristics of *Populus trichocarpa, Populus deltoides* and *Populus trichocarpa*× *P. deltoides* clones. Tree Physiol. 8, 145–159. 10.1093/treephys/8.2.145 14972886

[B4] Björkman O. (1981). Responses to different quantum flux densities, in Physiological Plant Ecology I, eds Lange O. L. Nobel P. S. Osmond C. B. Ziegler H. (Berlin; Heidelberg: Springer Press), 57–107.

[B5] Blankenship R. E. Tiede D. M. Barber J. Brudvig G. W. Fleming G. Ghirardi M.. (2011). Comparing photosynthetic and photovoltaic efficiencies and recognizing the potential for improvement. Science 332, 805–809. 10.1126/science.1200165 21566184

[B6] Boardman N. (1977). Comparative photosynthesis of sun and shade plants. Annu. Rev. Plant Physiol. 28, 355–377. 10.1146/annurev.pp.28.060177.002035

[B7] Brennan T. Frenkel C. (1977). Involvement of hydrogen peroxide in the regulation of senescence in pear. Plant Physiol. 59, 411–416. 10.1104/pp.59.3.411 16659863 PMC542414

[B8] Casal J. J. (2013). Photoreceptor signaling networks in plant responses to shade. Annu. Rev. Plant Physiol. 64, 403–427. 10.1146/annurev-arplant-050312-120221 23373700

[B9] Cope K. R. Bugbee B. (2013). Spectral effects of three types of white light-emitting diodes on plant growth and development: absolute versus relative amounts of blue light. Hortscience 48, 504–509.

[B10] Evans J. (1987). The dependence of quantum yield on wavelength and growth irradiance. Funct. Plant Biol. 14, 69–79. 10.1071/pp9870069

[B11] Fukuda N. Ajima C. Yukawa T. Olsen J. E. (2016). Antagonistic action of blue and red light on shoot elongation in petunia depends on gibberellin, but the effects on flowering are not generally linked to gibberellin. Environ. Exp. Bot. 121, 102–111. 10.1016/j.envexpbot.2015.06.014

[B12] Hernández R. Kubota C. (2016). Physiological responses of cucumber seedlings under different blue and red photon flux ratios using LEDs. Environ. Exp. Bot. 121, 66–74. 10.1016/j.envexpbot.2015.04.001

[B13] Hirai T. Amaki W. Watanabe H. (2006). Action of blue or red monochromatic light on stem internodal growth depends on plant species. Acta Hort. 711, 345–350. 10.17660/ActaHortic.2006.711.47

[B14] Hoffmann A. M. Noga G. Hunsche M. (2015). High blue light improves acclimation and photosynthetic recovery of pepper plants exposed to UV stress. Environ. Exp. Bot. 109, 254–263. 10.1016/j.envexpbot.2014.06.017

[B15] Hogewoning S. W. Trouwborst G. Maljaars H. Poorter H. Van Ieperen W. Harbinson J. (2010). Blue light dose-responses of leaf photosynthesis, morphology, and chemical composition of *Cucumis sativus* grown under different combinations of red and blue light. J. Exp. Bot. 61, 3107–3117. 10.1093/jxb/erq132 20504875 PMC2892149

[B16] Hogewoning S. W. Trouwborst G. Meinen E. Van Ieperen W. (2012). Finding the optimal growth-light spectrum for greenhouse crops. Acta Hort. 956, 357–363. 10.17660/ActaHortic.2012.956.41

[B17] Huber S. C. Huber J. L. (1996). Role and regulation of sucrose-phosphate synthase in higher plants. Annu. Rev. Plant Biol. 47, 431–444. 10.1146/annurev.arplant.47.1.431 15012296

[B18] Inoue S.-I. Takemiya A. Shimazaki K.-I. (2010). Phototropin signaling and stomatal opening as a model case. Curr. Opin. Plant Biol. 13, 587–593. 10.1016/j.pbi.2010.09.002 20920881

[B19] Johkan M. Shoji K. Goto F. Hashida S.-N. Yoshihara T. (2010). Blue light-emitting diode light irradiation of seedlings improves seedling quality and growth after transplanting in red leaf lettuce. Hortscience 45, 1809–1814.

[B20] Kazemi N. Khavari-Nejad R. A. Fahimi H. Saadatmand S. Nejad-Sattari T. (2010). Effects of exogenous salicylic acid and nitric oxide on lipid peroxidation and antioxidant enzyme activities in leaves of *Brassica napus* L. under nickel stress. Sci. Hort. Amsterdam 126, 402–407. 10.1016/j.scienta.2010.07.037

[B21] Kim Y. H. Lee M. G. (2004). Tuber production and growth of potato transplants grown under different light quality. Acta Hort. 659, 267–272. 10.17660/ActaHortic.2004.659.34

[B22] Lewis M. C. (1972). The physiological significance of variation in leaf structure. Sci. Prog. (1933-), 25–51. 26041397

[B23] Li H. Tang C. Xu Z. (2013). The effects of different light qualities on rapeseed (*Brassica napus* L.) plantlet growth and morphogenesis *in vitro*. Sci. Hort. Amsterdam 150, 117–124. 10.1016/j.scienta.2012.10.009

[B24] Li H. Xu Z. Tang C. (2010). Effect of light-emitting diodes on growth and morphogenesis of upland cotton (*Gossypium hirsutum* L.) plantlets *in vitro*. Plant Cell Tiss. Org. 103, 155–163. 10.1007/s11240-010-9763-z

[B25] Li J. G. Li G. Wang H. Y. Deng X. W. (2011). Phytochrome signaling mechanisms. Arabidopsis Book 3:e0148. 10.1199/tab.0148 22303272 PMC3268501

[B26] Lichtenthaler H. Buschmann C. Rahmsdorf U. (1980). The importance of blue light for the development of sun-type chloroplasts, in The Blue Light Syndrome, ed Senger H. (Berlin; Heidelberg: Springer Press), 485–494.

[B27] Long S. Bernacchi C. (2003). Gas exchange measurements, what can they tell us about the underlying limitations to photosynthesis? Procedures and sources of error. J. Exp. Bot. 54, 2393–2401. 10.1093/jxb/erg262 14512377

[B28] Loreto F. Tsonev T. Centritto M. (2009). The impact of blue light on leaf mesophyll conductance. J. Exp. Bot. 60, 2283–2290. 10.1093/jxb/erp112 19395388

[B29] Macedo A. F. Leal-Costa M. V. Tavares E. S. Lage C. L. S. Esquibel M. A. (2011). The effect of light quality on leaf production and development of *in vitro*-cultured plants of *Alternanthera brasiliana* Kuntze. Environ. Exp. Bot. 70, 43–50. 10.1016/j.envexpbot.2010.05.012

[B30] McCree K. J. (1972). Test of current definitions of photosynthetically active radiation against leaf photosynthesis data. Agric. Meteorol. 10, 443–453. 10.1016/0002-1571(72)90045-3

[B31] Nishiyama Y. Yamamoto H. Allakhverdiev S. I. Inaba M. Yokota A. Murata N. (2001). Oxidative stress inhibits the repair of photodamage to the photosynthetic machinery. EMBO J. 20, 5587–5594. 10.1093/emboj/20.20.5587 11598002 PMC125664

[B32] Ohnishi N. Allakhverdiev S. I. Takahashi S. Higashi S. Watanabe M. Nishiyama Y.. (2005). Two-step mechanism of photodamage to photosystem II: step 1 occurs at the oxygen-evolving complex and step 2 occurs at the photochemical reaction center. Biochemistry 44, 8494–8499. 10.1021/bi047518q 15938639

[B33] Rondanini D. P. Del Pilar Vilariño M. Roberts M. E. Polosa M. A. Botto J. F. (2014). Physiological responses of spring rapeseed (*Brassica napus*) to red/far-red ratios and irradiance during pre- and post- flowering stages. Physiol. Plantarum 152, 784–794. 10.1111/ppl.12227 24814241

[B34] Sæbø A. Krekling T. Appelgren M. (1995). Light quality affects photosynthesis and leaf anatomy of birch plantlets *in vitro*. Plant Cell Tissue Organ Cult. 41, 177–185. 10.1007/BF00051588

[B35] Sabzalian M. R. Heydarizadeh P. Zahedi M. Boroomand A. Agharokh M. Sahba M. R. (2014). High performance of vegetables, flowers, and medicinal plants in a red-blue LED incubator for indoor plant production. Agron. Sustain. Dev. 34, 879–886. 10.1007/s13593-014-0209-6

[B36] Schreiber U. (2004). Pulse-amplitude-modulation (PAM) fluorometry and saturation pulse method: an overview, in Chlorophyll a Fluorescence, ed George and Govindjee C. P. (Dordrecht: Springer Press), 279–319.

[B37] Schuerger A. C. Brown C. S. Stryjewski E. C. (1997). Anatomical features of pepper plants (*Capsicum annuum* L.) grown under red light-emitting diodes supplemented with blue or far-red light. Ann. Bot. Lond. 79, 273–282. 10.1006/anbo.1996.0341 11540425

[B38] Sellaro R. Crepy M. Trupkin S. A. Karayekov E. Buchovsky A. S. Rossi C.. (2010). Cryptochrome as a sensor of the blue/green ratio of natural radiation in *Arabidopsis*. Plant Physiol. 154, 401–409. 10.1104/pp.110.160820 20668058 PMC2938137

[B39] Sonoike K. (2011). Photoinhibition of photosystem I. Physiol. Plantarum 142, 56–64. 10.1111/j.1399-3054.2010.01437.x 21128947

[B40] Tenhaken R. (2015). Cell wall remodeling under abiotic stress. Front. Plant Sci. 5:771. 10.3389/fpls.2014.00771 25709610 PMC4285730

[B41] Terashima I. Hanba Y. T. Tazoe Y. Vyas P. Yano S. (2006). Irradiance and phenotype: comparative eco-development of sun and shade leaves in relation to photosynthetic CO_2_ diffusion. J. Exp. Bot. 57, 343–354. 10.1093/jxb/erj014 16356943

[B42] Terashima I. Hikosaka K. (1995). Comparative ecophysiology of leaf and canopy photosynthesis. Plant Cell Environ. 18, 1111–1128. 10.1111/j.1365-3040.1995.tb00623.x

[B43] Terashima I. Miyazawa S.-I. Hanba Y. T. (2001). Why are sun leaves thicker than shade leaves? Consideration based on analyses of CO_2_ diffusion in the leaf. J. Plant Res. 114, 93–105. 10.1007/PL00013972

[B44] Trouwborst G. Hogewoning S. W. Van Kooten O. Harbinson J. Van Ieperen W. (2016). Plasticity of photosynthesis after the “red light syndrome” in cucumber. Environ. Exp. Bot. 121, 75–82. 10.1016/j.envexpbot.2015.05.002

[B45] Upadhyaya A. Sankhla D. Davis T. D. Sankhla N. Smith B. (1985). Effect of paclobutrazol on the activities of some enzymes of activated oxygen metabolism and lipid peroxidation in senescing soybean leaves. J. Plant Physiol. 121, 453–461. 10.1016/S0176-1617(85)80081-X

[B46] Verma A. K. Upadhyay S. Verma P. C. Solomon S. Singh S. B. (2011). Functional analysis of sucrose phosphate synthase (SPS) and sucrose synthase (SS) in sugarcane (*Saccharum*) cultivars. Plant Biol. 13, 325–332. 10.1111/j.1438-8677.2010.00379.x 21309979

[B47] Wang Y. S. Yang Z. M. (2005). Nitric oxide reduces aluminum toxicity by preventing oxidative stress in the roots of *Cassia tora* L. Plant Cell Physiol. 46, 1915–1923. 10.1093/pcp/pci202 16179356

[B48] Yano S. Terashima I. (2001). Separate localization of light signal perception for sun or shade type chloroplast and palisade tissue differentiation in *Chenopodium album*. Plant Cell Physiol. 42, 1303–1310. 10.1093/pcp/pce183 11773522

[B49] Yu X. (1999). Sucrose synthetase and sucrose phosphate synthase extraction and assays, in Guide of Modern Plant Physiology Experiment, ed Tang Z. C. (Beijing, Science Press), 126.

[B50] Yu X. Liu H. Klejnot J. Lin C. (2010). The cryptochrome blue light receptors. Arabidopsis Book 8:e0135. 10.1199/tab.0135 21841916 PMC3155252

[B51] Zhao X. Yu X. Foo E. Symons G. M. Lopez J. Bendehakkalu K. T.. (2007). A study of gibberellin homeostasis and cryptochrome-mediated blue light inhibition of hypocotyl elongation. Plant Physiol. 145, 106–118. 10.1104/pp.107.099838 17644628 PMC1976579

